# *cis*-[Aqua/methanol(0.45/1.55)](1,1,1-trifluoro-5,5-dimethyl­hexane-2,4-dionato)nickel(II)–*cis*-[aqua/methanol(1.49/0.51)](1,1,1-trifluoro-5,5-dimethyl­hexane-2,4-dionato)nickel(II) (1/1)

**DOI:** 10.1107/S1600536809001846

**Published:** 2009-01-23

**Authors:** Gerald O. Hunter, Matthias Zeller, Brian D. Leskiw

**Affiliations:** a Youngstown State University, Department of Chemistry, 1 University Plaza, Youngstown, OH 44555, USA

## Abstract

The title compound, [Ni(C_8_H_10_F_3_O_2_)_2_(CH_4_O)_1.55_(H_2_O)_0.45_][Ni(C_8_H_10_F_3_O_2_)_2_(CH_4_O)_0.51_(H_2_O)_1.49_], is an octa­hedral nickel(II) complex with two acetyl­acetonate-like 1,1,1-trifluoro-5,5-dimethyl­hexane-2,4-dionate ligands. The two chelating ligands are in *cis* positions with respect to each other and the remaining two adjacent coordination sites are taken up by water and methanol donor mol­ecules. In both crystallographically independent mol­ecules, each donor site shows disorder of methanol and water with occupancies of 0.51 (1) and 0.55 (1) in favor of methanol. The remaining two donor sites are not disordered and are water for the first and methanol for the second independent mol­ecule. Rotational disorder is observed for one of the *tert*-butyl groups, the occupancy rate for the major component here is 0.687 (9). O—H⋯O hydrogen bonds connect the two independent mol­ecules with each other and, across a crystallographic inversion center, they are combined with two neighboring mol­ecules to form a centrosymmetric hydrogen-bonded tetra­mer.

## Related literature

For information regarding the synthesis of various metal β-diketonates refer to Watson & Lin (1966 ▶). For mass spectrometry-related articles, see: Lerach & Leskiw (2008 ▶); Schildcrout (1976 ▶). For a variety of applications and properties of metal β-diketonate complexes, see: Burtoloso (2005 ▶); Katok *et al.* (2006 ▶); Condorelli *et al.* (2007 ▶). Lerach *et al.* (2007 ▶) and Hunter *et al.* (2009 ▶) report the structures of Co- and Zn-complexes with the same ligand.
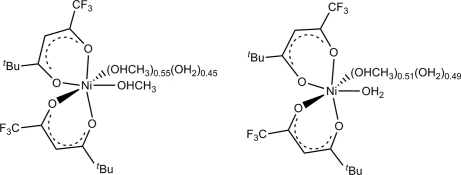


## Experimental

### 

#### Crystal data

[Ni(C_8_H_10_F_3_O_2_)_2_(CH_4_O)_1.55_(H_2_O)_0.45_][Ni(C_8_H_10_F_3_O_2_)_2_(CH_4_O)_0.51_(H_2_O)_1.49_]
*M* *_r_* = 998.89
Triclinic, 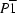

*a* = 11.327 (2) Å
*b* = 14.701 (3) Å
*c* = 15.432 (3) Å
α = 101.379 (3)°
β = 104.946 (3)°
γ = 107.678 (3)°
*V* = 2257.0 (8) Å^3^
*Z* = 2
Mo *K*α radiation
μ = 0.94 mm^−1^
*T* = 100 (2) K
0.20 × 0.20 × 0.15 mm

#### Data collection

Bruker SMART APEX CCD diffractometer
Absorption correction: multi-scan (*APEX2*; Bruker, 2008 ▶) *T*_min_ = 0.570, *T*_max_ = 0.869
22225 measured reflections
10979 independent reflections
5010 reflections with *I* > 2σ(*I*)
*R*_int_ = 0.075

#### Refinement

*R*[*F* ^2^ > 2σ(*F* ^2^)] = 0.057
*wR*(*F* ^2^) = 0.155
*S* = 1.00
10979 reflections
619 parameters
9 restraints
H atoms treated by a mixture of independent and constrained refinement
Δρ_max_ = 0.69 e Å^−3^
Δρ_min_ = −0.60 e Å^−3^

### 

Data collection: *APEX2* (Bruker, 2008 ▶); cell refinement: *SAINT* (Bruker, 2008 ▶); data reduction: *SAINT*; program(s) used to solve structure: *SHELXTL* (Sheldrick, 2008 ▶); program(s) used to refine structure: *SHELXTL*; molecular graphics: *SHELXTL*; software used to prepare material for publication: *SHELXTL*.

## Supplementary Material

Crystal structure: contains datablocks I, global. DOI: 10.1107/S1600536809001846/lx2082sup1.cif

Structure factors: contains datablocks I. DOI: 10.1107/S1600536809001846/lx2082Isup2.hkl

Additional supplementary materials:  crystallographic information; 3D view; checkCIF report

## Figures and Tables

**Table 1:** Hydrogen-bond geometry (Å, °)

*D*—H⋯*A*	*D*—H	H⋯*A*	*D*⋯*A*	*D*—H⋯*A*
O5—H5*A*⋯O9^i^	0.82 (4)	1.87 (2)	2.692 (4)	175 (5)
O6—H6*D*⋯O7	0.84 (4)	1.89 (2)	2.701 (4)	164 (5)
O6—H6*E*⋯O12^i^	0.81 (4)	2.16 (2)	2.951 (5)	164 (5)
O11—H11*A*⋯O1	0.85 (5)	1.93 (5)	2.764 (4)	166 (5)
O12—H12*A*⋯O3	0.84 (2)	1.94 (2)	2.778 (4)	173 (5)
O12—H12*B*⋯O10	0.84 (2)	2.5 (1)	2.861 (4)	108 (11)

Symmetry code: (i) 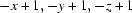.

**Fig. 1.**
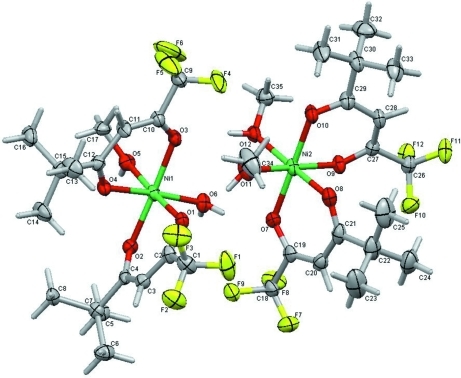
Thermal ellipsoid respresentation of the two crystallograpically independent molecules. The probability level for the anisotropic displacement parameters is at 50%. Minor moieties of disordered sections are omitted for clarity.

**Fig. 2.**
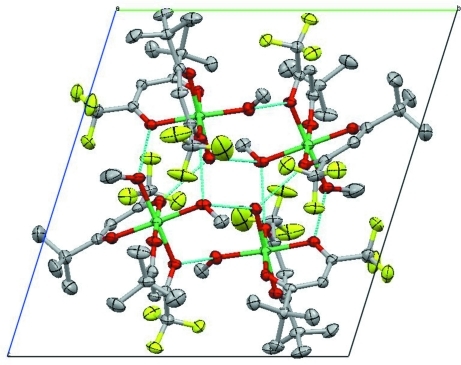
Packing view of the title compound along the [100]. Hydrogen bonds are indicated as dashed lines. Minor moieties of disordered moieties and hydrogen atoms are omitted for clarity.

## supplementary crystallographic information

### Comment

The research of metal β-diketonate compounds can be traced back to the early to mid 1950's. Since then extensive research has been conducted in order to gain a better understanding of these compounds for a wide variety of scientific applications. Some of the more recent applications are catalysis (Burtoloso, 2005), carbon-nanotube structures (Katok *et al.*, 2006), and the deposition of metallic or ceramic then films (Condorelli *et al.*, 2007). Our own research group is most interested in investigating gas-phase rearrangements of selected metal β-diketonate complexes *via* mass spectrometry. Our overall goal is to examine stability through fragmentation and to better understand ligand exchange reactions in the gas phase. Several acetylacetonate and substituted acetylacetonate species were already observed to undergo various ligand exchange reactions and association reactions (Schildcrout, 1976; Lerach & Leskiw, 2008). To completely characterize the complexes prior to their use in mass spectrometry, several solid state structures of such complexes have been determined including those of the Co-, Zn and Ni-complexes with the ligand 1,1,1-trifluoro-5,5-dimethylhexane-2,4-dionate. The Ni complex is the title complex of this report, the structures of the Co- and Zn complexes were reported recently (Lerach *et al.*, 2007; Hunter *et al.*, 2009).

The title compound, the Ni derivative of this series, has the general formula [Ni(O_2_C_8_F_3_)_2_*L*_2_] (*L* = H_2_O, HOCH_3_). It is an octahedral nickel(II) complex with two acetyl acetonate like 1,1,1-trifluoro-5,5-dimethylhexane-2,4-dionate ligands. The two chelating ligands are in *cis* position to each other and the remaining two adjacent coordination sites are taken up by water and methanol donor molecules (Figure 1). In both crystallographically independent molecules each one donor site shows disorder of methanol and water with occupancies of 0.51 (1) and 0.55 (1) in favor of methanol. The remaining two donor sites are not disordered and are water for the first and methanol for the second independent molecule. Rotational disorder is observed for one of the *tert*-butyl groups, the occupancy rate for the major component here is 0.687 (9).

Hydrogen bonds connect the two independent molecules with each other. Across a crystallographic inversion center, they are combined with two neighboring molecules to form a centrosymmetric hydrogen bonded [Ni(O_2_C_8_F_3_)_2_*L*_2_] tetramer (Figure 2).

### Experimental

The synthesis of the title compound was adapted from Watson & Lin (1966). 0.40 ml (2.5 mmol) of the ligand were added to a stirring solution of 0.25 g NiCl_2_ (1.97 mmol) and 50 ml of de-ionized water. Diluted 1:1 (*v*/*v*) NH_4_OH was added dropwise to the mixture until no more visible precipitate formed. The mixture was stirred overnight at room temperature, and the precipitate was isolated *via* vacuum filtration resulting in a pale blue powder. The powder was dried at room temperature overnight resulting in a blue-green product which was re-crystallized overnight by vapor diffusion of hexanes into a solution in diethyl ether.

### Refinement

Disorder is observed for one hydroxyl group and two of the methanol ligands are only partially present with the remainder taken up by water molecules. The occupancy ratio for the two *tert*-butyl moieties is 0.687 (9) to 0.313 (9). The rate of presence for the methanol molecules is 0.51 (1) and 0.55 (1), respectively.

OH hydrogen atoms were located in difference density Fourier maps and were refined with an O—H distance restraint of 0.84 (2) Å. H···H distances within disordered water molecules were restrained to 1.35 Å. All other H atoms were placed in calculated positions with C—H distances of 0.98 (methyl) and 0.95 Å (CH). The methyl and hydroxyl H's were refined with an isotropic displacement parameter *U*_iso_ of 1.5 times *U*_eq_ of the adjacent carbon or oxygen atom, and the C—H hydrogen atom with *U*_iso_ = 1.2 *U*_eq_(C). Methyl hydrogen atoms were allowed to rotate to best fit the experimental electron density.

### Crystal data

**Table:**

[Ni(C_8_H_10_F_3_O_2_)_2_(CH_4_O)_1.55_(H_2_O)_0.45_] [Ni(C_8_H_10_F_3_O_2_)_2_(CH_4_O)_0.51_(H_2_O)_1.49_]	*Z* = 2
*M**_r_* = 998.89	*F*(000) = 1032.9
Triclinic, *P*1	*D*_x_ = 1.470 Mg m^−^^3^
Hall symbol: -P 1	Mo *K*α radiation, λ = 0.71073 Å
*a* = 11.327 (2) Å	Cell parameters from 3507 reflections
*b* = 14.701 (3) Å	θ = 2.3–28.8°
*c* = 15.432 (3) Å	µ = 0.94 mm^−^^1^
α = 101.379 (3)°	*T* = 100 K
β = 104.946 (3)°	Block, green
γ = 107.678 (3)°	0.20 × 0.20 × 0.15 mm
*V* = 2257.0 (8) Å^3^	

### Data collection

**Table:**

Bruker SMART APEX CCD diffractometer	10979 independent reflections
Radiation source: fine-focus sealed tube	5010 reflections with *I* > 2σ(*I*)
graphite	*R*_int_ = 0.075
ω scans	θ_max_ = 28.3°, θ_min_ = 1.4°
Absorption correction: multi-scan (*APEX2*; Bruker, 2008)	*h* = −15→15
*T*_min_ = 0.570, *T*_max_ = 0.869	*k* = −19→19
22225 measured reflections	*l* = −20→20

### Refinement

**Table:**

Refinement on *F*^2^	Primary atom site location: structure-invariant direct methods
Least-squares matrix: full	Secondary atom site location: difference Fourier map
*R*[*F*^2^ > 2σ(*F*^2^)] = 0.057	Hydrogen site location: inferred from neighbouring sites
*wR*(*F*^2^) = 0.155	H atoms treated by a mixture of independent and constrained refinement
*S* = 1.00	*w* = 1/[σ^2^(*F*_o_^2^) + (0.0561*P*)^2^] where *P* = (*F*_o_^2^ + 2*F*_c_^2^)/3
10979 reflections	(Δ/σ)_max_ = 0.003
619 parameters	Δρ_max_ = 0.69 e Å^−^^3^
9 restraints	Δρ_min_ = −0.60 e Å^−^^3^

### Special details

**Table:**

Experimental. Disorder is observed for one hydroxyl group and two of the methanol ligands are only partially present with the remainder taken up by water molecules. The occupancy ratio for the two *tert*-butyl moieties is 0.687 (9) to 0.313 (9). The rate of presence for the methanol molecules is 0.51 (1) and 0.55 (1), respectively.OH hydrogen atoms were located in difference denisty Fourier maps and were refined with an O—H distance restraint of 0.84 (2) Å. The second hydrogen atoms in disordered water molecules were restrained to have a distance of 1.35 (2) Å from the position of the first H atom. All other H atoms were placed in calculated positions with C—H distances of 0.98 (methyl) and 0.95 Å (CH). The methyl and hydroxyl H's were refined with an isotropic displacement parameter *U*_iso_ of 1.5 times *U*_eq_ of the adjacent carbon or oxygen atom, and the C—H hydrogen atom with *U*_iso_ = 1.2 *U*_eq_(C). Methyl hydrogen atoms were allowed to rotate to best fit the experimental electron density.
Geometry. All e.s.d.'s (except the e.s.d. in the dihedral angle between two l.s. planes) are estimated using the full covariance matrix. The cell e.s.d.'s are taken into account individually in the estimation of e.s.d.'s in distances, angles and torsion angles; correlations between e.s.d.'s in cell parameters are only used when they are defined by crystal symmetry. An approximate (isotropic) treatment of cell e.s.d.'s is used for estimating e.s.d.'s involving l.s. planes.
Refinement. Refinement of *F*^2^ against ALL reflections. The weighted *R*-factor *wR* and goodness of fit *S* are based on *F*^2^, conventional *R*-factors *R* are based on *F*, with *F* set to zero for negative *F*^2^. The threshold expression of *F*^2^ > σ(*F*^2^) is used only for calculating *R*-factors(gt) *etc*. and is not relevant to the choice of reflections for refinement. *R*-factors based on *F*^2^ are statistically about twice as large as those based on *F*, and *R*- factors based on ALL data will be even larger.

### Fractional atomic coordinates and isotropic or equivalent isotropic displacement parameters (Å^2^)

**Table:**

	*x*	*y*	*z*	*U*_iso_*/*U*_eq_	Occ. (<1)
C1	0.2154 (5)	0.0370 (4)	0.2924 (4)	0.0382 (13)	
C2	0.2772 (4)	0.1381 (3)	0.2784 (3)	0.0271 (11)	
C3	0.3419 (4)	0.1443 (3)	0.2152 (3)	0.0262 (11)	
H3	0.3423	0.0836	0.1800	0.031*	
C4	0.4089 (4)	0.2337 (4)	0.1973 (3)	0.0227 (10)	
C5	0.4801 (4)	0.2298 (3)	0.1255 (3)	0.0275 (11)	
C6	0.5271 (5)	0.1424 (4)	0.1132 (4)	0.0398 (13)	
H6A	0.5862	0.1463	0.1738	0.060*	
H6B	0.5742	0.1458	0.0681	0.060*	
H6C	0.4509	0.0790	0.0896	0.060*	
C7	0.6002 (5)	0.3285 (4)	0.1560 (4)	0.0366 (13)	
H7A	0.5707	0.3849	0.1606	0.055*	
H7B	0.6448	0.3271	0.1095	0.055*	
H7C	0.6614	0.3362	0.2174	0.055*	
C8	0.3812 (5)	0.2206 (4)	0.0314 (3)	0.0324 (12)	
H8A	0.3035	0.1589	0.0127	0.049*	
H8B	0.4227	0.2187	−0.0169	0.049*	
H8C	0.3543	0.2783	0.0387	0.049*	
C9	−0.0415 (5)	0.3477 (5)	0.3914 (4)	0.0423 (14)	
C10	0.0368 (5)	0.3223 (4)	0.3293 (3)	0.0282 (11)	
C11	−0.0341 (4)	0.2624 (4)	0.2390 (3)	0.0319 (12)	
H11	−0.1273	0.2345	0.2219	0.038*	
C12	0.0219 (4)	0.2388 (3)	0.1682 (3)	0.0255 (11)	
C13	−0.0694 (5)	0.1722 (4)	0.0695 (3)	0.0321 (12)	
C14	0.0128 (5)	0.1427 (4)	0.0125 (3)	0.0442 (14)	
H14A	0.0772	0.2032	0.0099	0.066*	
H14B	−0.0453	0.1014	−0.0514	0.066*	
H14C	0.0591	0.1046	0.0426	0.066*	
C15	−0.1688 (5)	0.0761 (4)	0.0745 (4)	0.0431 (14)	
H15A	−0.2250	0.0332	0.0107	0.065*	
H15B	−0.2237	0.0942	0.1093	0.065*	
H15C	−0.1209	0.0402	0.1067	0.065*	
C16	−0.1424 (5)	0.2326 (4)	0.0233 (3)	0.0419 (14)	
H16A	−0.0781	0.2943	0.0226	0.063*	
H16B	−0.1966	0.2495	0.0593	0.063*	
H16C	−0.1989	0.1926	−0.0413	0.063*	
C17	0.2152 (9)	0.5096 (8)	0.2473 (7)	0.033 (3)	0.508 (12)
H17A	0.1703	0.4709	0.1808	0.050*	0.508 (12)
H17B	0.2522	0.5809	0.2530	0.050*	0.508 (12)
H17C	0.1521	0.4986	0.2809	0.050*	0.508 (12)
C18	0.6284 (5)	0.2615 (4)	0.5218 (3)	0.0374 (13)	
C19	0.5238 (5)	0.2489 (4)	0.5689 (3)	0.0295 (11)	
C20	0.5083 (5)	0.1814 (4)	0.6188 (3)	0.0332 (12)	
H20	0.5686	0.1481	0.6259	0.040*	
C21	0.4099 (5)	0.1568 (4)	0.6610 (3)	0.0343 (12)	
C22	0.4030 (6)	0.0779 (4)	0.7129 (4)	0.0468 (15)	
C23	0.3917 (8)	−0.0193 (4)	0.6472 (4)	0.073 (2)	
H23A	0.3114	−0.0435	0.5922	0.110*	
H23B	0.3880	−0.0698	0.6805	0.110*	
H23C	0.4686	−0.0069	0.6267	0.110*	
C24	0.5299 (6)	0.1188 (4)	0.7993 (4)	0.0576 (17)	
H24A	0.6068	0.1340	0.7787	0.086*	
H24B	0.5297	0.0685	0.8326	0.086*	
H24C	0.5341	0.1798	0.8415	0.086*	
C25	0.2841 (7)	0.0601 (5)	0.7459 (4)	0.0661 (19)	
H25A	0.2927	0.1229	0.7881	0.099*	
H25B	0.2800	0.0098	0.7795	0.099*	
H25C	0.2033	0.0363	0.6914	0.099*	
C26	0.5696 (5)	0.4356 (4)	0.8933 (3)	0.0320 (12)	
C27	0.4424 (4)	0.4033 (3)	0.8111 (3)	0.0251 (11)	
C28	0.3258 (4)	0.3757 (3)	0.8271 (3)	0.0291 (11)	
H28	0.3273	0.3768	0.8891	0.035*	
C29	0.2011 (5)	0.3452 (4)	0.7550 (3)	0.0302 (11)	
C30	0.0798 (5)	0.3395 (4)	0.7834 (3)	0.0377 (13)	
C31	−0.0429 (8)	0.2896 (9)	0.6985 (6)	0.054 (3)	0.687 (9)
H31A	−0.1201	0.2843	0.7177	0.081*	0.687 (9)
H31B	−0.0487	0.2225	0.6679	0.081*	0.687 (9)
H31C	−0.0399	0.3293	0.6545	0.081*	0.687 (9)
C32	0.0967 (8)	0.4400 (7)	0.8317 (7)	0.053 (3)	0.687 (9)
H32A	0.1105	0.4826	0.7909	0.080*	0.687 (9)
H32B	0.1734	0.4668	0.8896	0.080*	0.687 (9)
H32C	0.0178	0.4386	0.8473	0.080*	0.687 (9)
C33	0.0716 (8)	0.2709 (7)	0.8505 (6)	0.048 (3)	0.687 (9)
H33A	0.1459	0.3047	0.9098	0.073*	0.687 (9)
H33B	0.0753	0.2071	0.8201	0.073*	0.687 (9)
H33C	−0.0113	0.2584	0.8633	0.073*	0.687 (9)
C31B	0.0110 (18)	0.4102 (13)	0.7309 (13)	0.052 (6)	0.313 (9)
H31D	−0.0289	0.3769	0.6631	0.078*	0.313 (9)
H31E	0.0790	0.4757	0.7429	0.078*	0.313 (9)
H31F	−0.0569	0.4191	0.7564	0.078*	0.313 (9)
C32B	0.1036 (17)	0.3959 (16)	0.8881 (12)	0.050 (6)	0.313 (9)
H32D	0.0189	0.3915	0.8956	0.076*	0.313 (9)
H32E	0.1600	0.4664	0.9033	0.076*	0.313 (9)
H32F	0.1467	0.3650	0.9305	0.076*	0.313 (9)
C33B	−0.017 (2)	0.2381 (15)	0.746 (2)	0.091 (12)	0.313 (9)
H33D	0.0248	0.1920	0.7636	0.136*	0.313 (9)
H33E	−0.0524	0.2204	0.6771	0.136*	0.313 (9)
H33F	−0.0888	0.2333	0.7712	0.136*	0.313 (9)
C34	0.0684 (6)	0.1373 (5)	0.4794 (4)	0.069 (2)	
H34A	0.0956	0.0816	0.4885	0.104*	
H34B	0.0012	0.1164	0.4172	0.104*	
H34C	0.0320	0.1569	0.5279	0.104*	
C35	0.2792 (10)	0.5035 (7)	0.6105 (7)	0.038 (3)	0.546 (15)
H35A	0.1921	0.4725	0.6149	0.057*	0.546 (15)
H35B	0.2785	0.5554	0.5794	0.057*	0.546 (15)
H35C	0.3453	0.5335	0.6738	0.057*	0.546 (15)
F1	0.2653 (4)	0.0395 (2)	0.3817 (2)	0.0601 (10)	
F2	0.2309 (3)	−0.0384 (2)	0.2390 (2)	0.0493 (8)	
F3	0.0859 (3)	0.0125 (2)	0.2729 (2)	0.0579 (9)	
F4	0.0066 (3)	0.3460 (3)	0.4771 (2)	0.0767 (13)	
F5	−0.1676 (3)	0.2897 (3)	0.3588 (2)	0.0867 (14)	
F6	−0.0380 (5)	0.4389 (4)	0.3985 (3)	0.1089 (18)	
F7	0.7002 (3)	0.2064 (2)	0.5379 (2)	0.0514 (9)	
F8	0.7119 (3)	0.3569 (2)	0.5511 (2)	0.0502 (8)	
F9	0.5724 (3)	0.2372 (2)	0.42870 (19)	0.0478 (8)	
F10	0.6442 (3)	0.3849 (2)	0.8754 (2)	0.0463 (8)	
F11	0.5512 (3)	0.4239 (2)	0.97410 (18)	0.0427 (8)	
F12	0.6426 (3)	0.53284 (19)	0.91291 (18)	0.0367 (7)	
Ni1	0.28898 (6)	0.34142 (4)	0.30511 (4)	0.02390 (16)	
Ni2	0.32213 (6)	0.31408 (5)	0.61117 (4)	0.02661 (17)	
O1	0.2590 (3)	0.2076 (2)	0.3312 (2)	0.0269 (7)	
O2	0.4093 (3)	0.3171 (2)	0.2352 (2)	0.0236 (7)	
O3	0.1611 (3)	0.3641 (2)	0.3708 (2)	0.0265 (7)	
O4	0.1419 (3)	0.2711 (2)	0.1825 (2)	0.0271 (8)	
O5	0.3153 (3)	0.4793 (2)	0.2856 (2)	0.0305 (8)	
H5A	0.382 (3)	0.511 (3)	0.277 (3)	0.046*	
H5B	0.311 (7)	0.509 (5)	0.336 (4)	0.046*	0.492 (12)
O6	0.4469 (3)	0.4085 (2)	0.4268 (2)	0.0300 (8)	
H6D	0.455 (5)	0.369 (3)	0.458 (3)	0.045*	
H6E	0.518 (3)	0.442 (3)	0.426 (4)	0.045*	
O7	0.4599 (3)	0.3051 (2)	0.5532 (2)	0.0271 (7)	
O8	0.3270 (3)	0.1969 (2)	0.6578 (2)	0.0325 (8)	
O9	0.4634 (3)	0.4058 (2)	0.7340 (2)	0.0274 (7)	
O10	0.1850 (3)	0.3252 (3)	0.6687 (2)	0.0335 (8)	
O11	0.1784 (3)	0.2192 (3)	0.4861 (2)	0.0349 (9)	
H11A	0.205 (5)	0.206 (4)	0.440 (3)	0.052*	
O12	0.3086 (3)	0.4347 (3)	0.5613 (2)	0.0301 (8)	
H12A	0.260 (4)	0.416 (4)	0.5048 (16)	0.045*	
H12B	0.266 (8)	0.454 (10)	0.593 (4)	0.045*	0.454 (15)

### Atomic displacement parameters (Å^2^)

**Table:**

	*U*^11^	*U*^22^	*U*^33^	*U*^12^	*U*^13^	*U*^23^
C1	0.045 (3)	0.029 (3)	0.039 (3)	0.008 (3)	0.021 (3)	0.007 (3)
C2	0.026 (3)	0.025 (3)	0.021 (3)	0.002 (2)	0.004 (2)	0.003 (2)
C3	0.026 (3)	0.023 (3)	0.026 (3)	0.008 (2)	0.007 (2)	0.005 (2)
C4	0.018 (2)	0.031 (3)	0.017 (2)	0.008 (2)	0.0034 (18)	0.008 (2)
C5	0.026 (3)	0.026 (3)	0.028 (3)	0.007 (2)	0.009 (2)	0.006 (2)
C6	0.039 (3)	0.041 (3)	0.053 (4)	0.020 (3)	0.026 (3)	0.021 (3)
C7	0.031 (3)	0.036 (3)	0.042 (3)	0.010 (2)	0.015 (2)	0.014 (3)
C8	0.037 (3)	0.042 (3)	0.023 (3)	0.017 (2)	0.012 (2)	0.014 (2)
C9	0.036 (3)	0.065 (4)	0.025 (3)	0.024 (3)	0.010 (2)	0.005 (3)
C10	0.030 (3)	0.032 (3)	0.021 (3)	0.010 (2)	0.009 (2)	0.006 (2)
C11	0.016 (2)	0.046 (3)	0.022 (3)	0.005 (2)	0.002 (2)	0.001 (2)
C12	0.023 (3)	0.027 (3)	0.025 (3)	0.008 (2)	0.007 (2)	0.009 (2)
C13	0.024 (3)	0.037 (3)	0.026 (3)	0.008 (2)	0.005 (2)	0.002 (2)
C14	0.034 (3)	0.055 (4)	0.026 (3)	0.010 (3)	0.005 (2)	−0.009 (3)
C15	0.033 (3)	0.036 (3)	0.040 (3)	0.000 (2)	0.007 (2)	−0.006 (3)
C16	0.034 (3)	0.059 (4)	0.024 (3)	0.014 (3)	0.004 (2)	0.007 (3)
C17	0.034 (6)	0.043 (7)	0.040 (6)	0.024 (5)	0.022 (5)	0.020 (5)
C18	0.037 (3)	0.045 (4)	0.024 (3)	0.017 (3)	0.006 (2)	−0.001 (3)
C19	0.028 (3)	0.030 (3)	0.018 (2)	0.008 (2)	−0.001 (2)	−0.003 (2)
C20	0.039 (3)	0.028 (3)	0.027 (3)	0.015 (2)	0.003 (2)	0.005 (2)
C21	0.042 (3)	0.026 (3)	0.023 (3)	0.007 (3)	0.000 (2)	0.005 (2)
C22	0.069 (4)	0.031 (3)	0.036 (3)	0.014 (3)	0.012 (3)	0.015 (3)
C23	0.128 (7)	0.036 (4)	0.052 (4)	0.027 (4)	0.026 (4)	0.017 (3)
C24	0.081 (5)	0.052 (4)	0.042 (4)	0.031 (4)	0.012 (3)	0.022 (3)
C25	0.086 (5)	0.054 (4)	0.060 (4)	0.011 (4)	0.029 (4)	0.039 (4)
C26	0.034 (3)	0.031 (3)	0.025 (3)	0.010 (2)	0.005 (2)	0.008 (2)
C27	0.026 (3)	0.024 (3)	0.023 (3)	0.008 (2)	0.005 (2)	0.008 (2)
C28	0.032 (3)	0.033 (3)	0.017 (2)	0.006 (2)	0.006 (2)	0.008 (2)
C29	0.026 (3)	0.033 (3)	0.027 (3)	0.004 (2)	0.008 (2)	0.010 (2)
C30	0.029 (3)	0.049 (4)	0.023 (3)	0.003 (3)	0.007 (2)	0.007 (3)
C31	0.024 (4)	0.098 (9)	0.035 (5)	0.020 (5)	0.008 (4)	0.018 (5)
C32	0.036 (5)	0.064 (7)	0.059 (7)	0.021 (5)	0.020 (5)	0.006 (5)
C33	0.039 (5)	0.070 (7)	0.040 (5)	0.011 (5)	0.021 (4)	0.028 (5)
C31B	0.047 (12)	0.037 (12)	0.056 (13)	−0.001 (9)	0.023 (10)	0.002 (10)
C32B	0.031 (10)	0.080 (17)	0.028 (10)	0.008 (10)	0.016 (8)	0.001 (10)
C33B	0.060 (17)	0.029 (12)	0.18 (4)	−0.006 (11)	0.08 (2)	0.017 (16)
C34	0.055 (4)	0.075 (5)	0.042 (4)	−0.010 (4)	0.016 (3)	−0.003 (3)
C35	0.035 (6)	0.038 (7)	0.034 (6)	0.013 (5)	0.008 (4)	0.003 (5)
F1	0.100 (3)	0.0395 (19)	0.0349 (19)	0.0108 (19)	0.0235 (18)	0.0205 (16)
F2	0.068 (2)	0.0258 (17)	0.062 (2)	0.0143 (16)	0.0375 (18)	0.0131 (16)
F3	0.045 (2)	0.0343 (19)	0.090 (3)	0.0014 (15)	0.0344 (19)	0.0168 (18)
F4	0.056 (2)	0.168 (4)	0.0325 (19)	0.060 (3)	0.0294 (17)	0.038 (2)
F5	0.030 (2)	0.154 (4)	0.049 (2)	0.023 (2)	0.0169 (17)	−0.012 (2)
F6	0.194 (5)	0.098 (4)	0.119 (4)	0.104 (4)	0.117 (4)	0.048 (3)
F7	0.0467 (19)	0.066 (2)	0.053 (2)	0.0380 (18)	0.0171 (16)	0.0152 (18)
F8	0.0364 (18)	0.046 (2)	0.061 (2)	0.0073 (16)	0.0215 (16)	0.0067 (17)
F9	0.0466 (19)	0.074 (2)	0.0277 (17)	0.0309 (17)	0.0138 (14)	0.0108 (16)
F10	0.0430 (19)	0.051 (2)	0.0408 (18)	0.0281 (16)	0.0013 (14)	0.0044 (16)
F11	0.0382 (17)	0.054 (2)	0.0238 (15)	0.0046 (15)	0.0023 (13)	0.0165 (15)
F12	0.0326 (16)	0.0282 (16)	0.0317 (16)	−0.0006 (13)	0.0002 (12)	0.0048 (13)
Ni1	0.0211 (3)	0.0256 (3)	0.0187 (3)	0.0038 (3)	0.0044 (2)	0.0041 (3)
Ni2	0.0253 (3)	0.0307 (4)	0.0181 (3)	0.0052 (3)	0.0053 (3)	0.0063 (3)
O1	0.0255 (18)	0.0307 (19)	0.0201 (17)	0.0065 (15)	0.0060 (14)	0.0071 (15)
O2	0.0221 (17)	0.0204 (18)	0.0272 (18)	0.0050 (14)	0.0090 (14)	0.0088 (15)
O3	0.0221 (18)	0.0328 (19)	0.0189 (17)	0.0059 (15)	0.0051 (14)	0.0055 (15)
O4	0.0200 (18)	0.037 (2)	0.0209 (17)	0.0061 (15)	0.0068 (14)	0.0082 (15)
O5	0.029 (2)	0.031 (2)	0.034 (2)	0.0089 (16)	0.0150 (16)	0.0132 (17)
O6	0.0271 (19)	0.030 (2)	0.0263 (19)	0.0019 (16)	0.0070 (16)	0.0115 (16)
O7	0.0285 (18)	0.0284 (19)	0.0253 (18)	0.0112 (16)	0.0095 (14)	0.0082 (15)
O8	0.038 (2)	0.030 (2)	0.0233 (18)	0.0061 (17)	0.0089 (15)	0.0078 (16)
O9	0.0213 (17)	0.0322 (19)	0.0199 (17)	0.0005 (14)	0.0054 (13)	0.0065 (15)
O10	0.0275 (19)	0.049 (2)	0.0188 (18)	0.0099 (16)	0.0060 (14)	0.0096 (17)
O11	0.032 (2)	0.037 (2)	0.0220 (19)	−0.0012 (16)	0.0106 (15)	0.0025 (16)
O12	0.0267 (19)	0.039 (2)	0.0202 (18)	0.0113 (16)	0.0055 (15)	0.0049 (17)

### Geometric parameters (Å, °)

**Table:**

C1—F2	1.332 (6)	C24—H24C	0.9800
C1—F3	1.334 (6)	C25—H25A	0.9800
C1—F1	1.337 (6)	C25—H25B	0.9800
C1—C2	1.522 (6)	C25—H25C	0.9800
C2—O1	1.279 (5)	C26—F10	1.330 (6)
C2—C3	1.364 (6)	C26—F12	1.342 (5)
C3—C4	1.424 (6)	C26—F11	1.350 (5)
C3—H3	0.9500	C26—C27	1.522 (6)
C4—O2	1.247 (5)	C27—O9	1.278 (5)
C4—C5	1.530 (6)	C27—C28	1.361 (6)
C5—C6	1.531 (6)	C28—C29	1.429 (6)
C5—C8	1.540 (6)	C28—H28	0.9500
C5—C7	1.542 (6)	C29—O10	1.256 (5)
C6—H6A	0.9800	C29—C30	1.531 (7)
C6—H6B	0.9800	C30—C32	1.447 (10)
C6—H6C	0.9800	C30—C33B	1.45 (2)
C7—H7A	0.9800	C30—C31	1.499 (9)
C7—H7B	0.9800	C30—C32B	1.575 (17)
C7—H7C	0.9800	C30—C33	1.582 (9)
C8—H8A	0.9800	C30—C31B	1.68 (2)
C8—H8B	0.9800	C31—H31A	0.9800
C8—H8C	0.9800	C31—H31B	0.9800
C9—F4	1.302 (6)	C31—H31C	0.9800
C9—F6	1.310 (7)	C32—H32A	0.9800
C9—F5	1.319 (6)	C32—H32B	0.9800
C9—C10	1.532 (7)	C32—H32C	0.9800
C10—O3	1.274 (5)	C33—H33A	0.9800
C10—C11	1.368 (6)	C33—H33B	0.9800
C11—C12	1.434 (6)	C33—H33C	0.9800
C11—H11	0.9500	C31B—H31D	0.9800
C12—O4	1.239 (5)	C31B—H31E	0.9800
C12—C13	1.527 (6)	C31B—H31F	0.9800
C13—C14	1.531 (6)	C32B—H32D	0.9800
C13—C16	1.540 (7)	C32B—H32E	0.9800
C13—C15	1.547 (6)	C32B—H32F	0.9800
C14—H14A	0.9800	C33B—H33D	0.9800
C14—H14B	0.9800	C33B—H33E	0.9800
C14—H14C	0.9800	C33B—H33F	0.9800
C15—H15A	0.9800	C34—O11	1.409 (6)
C15—H15B	0.9800	C34—H34A	0.9800
C15—H15C	0.9800	C34—H34B	0.9800
C16—H16A	0.9800	C34—H34C	0.9800
C16—H16B	0.9800	C35—O12	1.320 (10)
C16—H16C	0.9800	C35—H35A	0.9800
C17—O5	1.377 (9)	C35—H35B	0.9800
C17—H17A	0.9800	C35—H35C	0.9800
C17—H17B	0.9800	C35—H12B	0.67 (13)
C17—H17C	0.9800	Ni1—O4	1.995 (3)
C18—F7	1.326 (6)	Ni1—O2	2.013 (3)
C18—F9	1.336 (5)	Ni1—O1	2.032 (3)
C18—F8	1.337 (6)	Ni1—O3	2.039 (3)
C18—C19	1.526 (7)	Ni1—O6	2.040 (3)
C19—O7	1.273 (5)	Ni1—O5	2.051 (3)
C19—C20	1.369 (6)	Ni2—O8	2.004 (3)
C20—C21	1.421 (7)	Ni2—O10	2.010 (3)
C20—H20	0.9500	Ni2—O7	2.013 (3)
C21—O8	1.248 (6)	Ni2—O9	2.022 (3)
C21—C22	1.528 (7)	Ni2—O11	2.063 (3)
C22—C23	1.528 (8)	Ni2—O12	2.098 (4)
C22—C25	1.528 (8)	O5—H5A	0.82 (4)
C22—C24	1.542 (8)	O5—H5B	0.83 (2)
C23—H23A	0.9800	O6—H6D	0.84 (4)
C23—H23B	0.9800	O6—H6E	0.81 (4)
C23—H23C	0.9800	O11—H11A	0.85 (5)
C24—H24A	0.9800	O12—H12A	0.838 (19)
C24—H24B	0.9800	O12—H12B	0.84 (2)
			
F2—C1—F3	106.6 (4)	C22—C25—H25C	109.5
F2—C1—F1	107.1 (4)	H25A—C25—H25C	109.5
F3—C1—F1	106.7 (4)	H25B—C25—H25C	109.5
F2—C1—C2	114.4 (4)	F10—C26—F12	106.8 (4)
F3—C1—C2	110.6 (4)	F10—C26—F11	106.5 (4)
F1—C1—C2	111.0 (4)	F12—C26—F11	105.9 (4)
O1—C2—C3	129.0 (4)	F10—C26—C27	112.0 (4)
O1—C2—C1	111.7 (4)	F12—C26—C27	111.2 (4)
C3—C2—C1	119.3 (4)	F11—C26—C27	114.0 (4)
C2—C3—C4	125.7 (4)	O9—C27—C28	129.1 (4)
C2—C3—H3	117.1	O9—C27—C26	112.1 (4)
C4—C3—H3	117.1	C28—C27—C26	118.8 (4)
O2—C4—C3	123.0 (4)	C27—C28—C29	123.1 (4)
O2—C4—C5	116.8 (4)	C27—C28—H28	118.5
C3—C4—C5	120.1 (4)	C29—C28—H28	118.5
C4—C5—C6	113.8 (4)	O10—C29—C28	123.9 (4)
C4—C5—C8	106.3 (4)	O10—C29—C30	117.3 (4)
C6—C5—C8	109.5 (4)	C28—C29—C30	118.7 (4)
C4—C5—C7	109.1 (4)	C32—C30—C33B	141.5 (10)
C6—C5—C7	108.7 (4)	C32—C30—C31	112.8 (7)
C8—C5—C7	109.2 (4)	C32—C30—C29	108.1 (5)
C5—C6—H6A	109.5	C33B—C30—C29	110.1 (10)
C5—C6—H6B	109.5	C31—C30—C29	110.1 (5)
H6A—C6—H6B	109.5	C33B—C30—C32B	116.1 (14)
C5—C6—H6C	109.5	C29—C30—C32B	117.6 (7)
H6A—C6—H6C	109.5	C32—C30—C33	111.1 (6)
H6B—C6—H6C	109.5	C31—C30—C33	107.3 (6)
C5—C7—H7A	109.5	C29—C30—C33	107.4 (5)
C5—C7—H7B	109.5	C33B—C30—C31B	106.6 (15)
H7A—C7—H7B	109.5	C29—C30—C31B	106.6 (7)
C5—C7—H7C	109.5	C30—C31—H31A	109.5
H7A—C7—H7C	109.5	C30—C31—H31B	109.5
H7B—C7—H7C	109.5	C30—C31—H31C	109.5
C5—C8—H8A	109.5	C30—C32—H32A	109.5
C5—C8—H8B	109.5	C30—C32—H32B	109.5
H8A—C8—H8B	109.5	C30—C32—H32C	109.5
C5—C8—H8C	109.5	C30—C33—H33A	109.5
H8A—C8—H8C	109.5	C30—C33—H33B	109.5
H8B—C8—H8C	109.5	C30—C33—H33C	109.5
F4—C9—F6	105.9 (5)	C30—C31B—H31D	109.5
F4—C9—F5	107.4 (5)	C30—C31B—H31E	109.5
F6—C9—F5	105.7 (5)	H31D—C31B—H31E	109.5
F4—C9—C10	113.0 (4)	C30—C31B—H31F	109.5
F6—C9—C10	109.8 (5)	H31D—C31B—H31F	109.5
F5—C9—C10	114.4 (4)	H31E—C31B—H31F	109.5
O3—C10—C11	130.0 (4)	C30—C32B—H32D	109.5
O3—C10—C9	113.0 (4)	C30—C32B—H32E	109.5
C11—C10—C9	116.9 (4)	H32D—C32B—H32E	109.5
C10—C11—C12	124.7 (4)	C30—C32B—H32F	109.5
C10—C11—H11	117.6	H32D—C32B—H32F	109.5
C12—C11—H11	117.6	H32E—C32B—H32F	109.5
O4—C12—C11	123.3 (4)	C30—C33B—H33D	109.5
O4—C12—C13	117.5 (4)	C30—C33B—H33E	109.5
C11—C12—C13	119.2 (4)	H33D—C33B—H33E	109.5
C12—C13—C14	109.3 (4)	C30—C33B—H33F	109.5
C12—C13—C16	107.8 (4)	H33D—C33B—H33F	109.5
C14—C13—C16	110.1 (4)	H33E—C33B—H33F	109.5
C12—C13—C15	110.3 (4)	O11—C34—H34A	109.5
C14—C13—C15	108.8 (4)	O11—C34—H34B	109.5
C16—C13—C15	110.4 (4)	H34A—C34—H34B	109.5
C13—C14—H14A	109.5	O11—C34—H34C	109.5
C13—C14—H14B	109.5	H34A—C34—H34C	109.5
H14A—C14—H14B	109.5	H34B—C34—H34C	109.5
C13—C14—H14C	109.5	O12—C35—H35A	109.5
H14A—C14—H14C	109.5	O12—C35—H35B	109.5
H14B—C14—H14C	109.5	O12—C35—H35C	109.5
C13—C15—H15A	109.5	O4—Ni1—O2	86.61 (12)
C13—C15—H15B	109.5	O4—Ni1—O1	88.71 (13)
H15A—C15—H15B	109.5	O2—Ni1—O1	90.57 (12)
C13—C15—H15C	109.5	O4—Ni1—O3	91.10 (12)
H15A—C15—H15C	109.5	O2—Ni1—O3	177.68 (12)
H15B—C15—H15C	109.5	O1—Ni1—O3	89.75 (12)
C13—C16—H16A	109.5	O4—Ni1—O6	176.00 (14)
C13—C16—H16B	109.5	O2—Ni1—O6	89.65 (13)
H16A—C16—H16B	109.5	O1—Ni1—O6	89.89 (13)
C13—C16—H16C	109.5	O3—Ni1—O6	92.65 (13)
H16A—C16—H16C	109.5	O4—Ni1—O5	92.70 (13)
H16B—C16—H16C	109.5	O2—Ni1—O5	92.81 (12)
O5—C17—H17A	109.5	O1—Ni1—O5	176.41 (13)
O5—C17—H17B	109.5	O3—Ni1—O5	86.92 (12)
O5—C17—H17C	109.5	O6—Ni1—O5	88.91 (13)
H17A—C17—H5B	140.5	O8—Ni2—O10	89.86 (14)
H17B—C17—H5B	99.1	O8—Ni2—O7	91.07 (13)
H17C—C17—H5B	84.8	O10—Ni2—O7	179.05 (14)
F7—C18—F9	107.1 (4)	O8—Ni2—O9	88.98 (13)
F7—C18—F8	107.1 (4)	O10—Ni2—O9	89.01 (13)
F9—C18—F8	107.1 (4)	O7—Ni2—O9	90.81 (13)
F7—C18—C19	114.2 (4)	O8—Ni2—O11	90.45 (13)
F9—C18—C19	110.4 (4)	O10—Ni2—O11	91.16 (13)
F8—C18—C19	110.6 (4)	O7—Ni2—O11	89.03 (13)
O7—C19—C20	128.9 (5)	O9—Ni2—O11	179.40 (14)
O7—C19—C18	111.7 (4)	O8—Ni2—O12	177.62 (13)
C20—C19—C18	119.4 (5)	O10—Ni2—O12	88.24 (13)
C19—C20—C21	125.7 (5)	O7—Ni2—O12	90.84 (13)
C19—C20—H20	117.2	O9—Ni2—O12	92.41 (13)
C21—C20—H20	117.2	O11—Ni2—O12	88.17 (13)
O8—C21—C20	122.8 (4)	C2—O1—Ni1	120.1 (3)
O8—C21—C22	117.5 (5)	C4—O2—Ni1	125.9 (3)
C20—C21—C22	119.7 (5)	C10—O3—Ni1	121.3 (3)
C21—C22—C23	109.4 (4)	C12—O4—Ni1	128.1 (3)
C21—C22—C25	109.6 (5)	C17—O5—Ni1	125.0 (5)
C23—C22—C25	110.3 (5)	C17—O5—H5A	109 (4)
C21—C22—C24	107.7 (4)	Ni1—O5—H5A	121 (4)
C23—C22—C24	110.7 (5)	Ni1—O5—H5B	100 (6)
C25—C22—C24	109.1 (5)	H5A—O5—H5B	111 (3)
C22—C23—H23A	109.5	Ni1—O6—H6D	112 (4)
C22—C23—H23B	109.5	Ni1—O6—H6E	120 (4)
H23A—C23—H23B	109.5	H6D—O6—H6E	112 (5)
C22—C23—H23C	109.5	C19—O7—Ni2	122.4 (3)
H23A—C23—H23C	109.5	C21—O8—Ni2	127.8 (3)
H23B—C23—H23C	109.5	C27—O9—Ni2	119.3 (3)
C22—C24—H24A	109.5	C29—O10—Ni2	125.7 (3)
C22—C24—H24B	109.5	C34—O11—Ni2	124.2 (3)
H24A—C24—H24B	109.5	C34—O11—H11A	111 (4)
C22—C24—H24C	109.5	Ni2—O11—H11A	116 (4)
H24A—C24—H24C	109.5	C35—O12—Ni2	118.8 (5)
H24B—C24—H24C	109.5	C35—O12—H12A	110 (4)
C22—C25—H25A	109.5	Ni2—O12—H12A	112 (4)
C22—C25—H25B	109.5	Ni2—O12—H12B	99 (9)
H25A—C25—H25B	109.5	H12A—O12—H12B	107 (3)
			
F2—C1—C2—O1	179.9 (4)	C28—C29—C30—C32B	17.2 (12)
F3—C1—C2—O1	59.5 (5)	O10—C29—C30—C33	128.3 (6)
F1—C1—C2—O1	−58.8 (6)	C28—C29—C30—C33	−53.7 (7)
F2—C1—C2—C3	−0.2 (7)	O10—C29—C30—C31B	−52.3 (8)
F3—C1—C2—C3	−120.6 (5)	C28—C29—C30—C31B	125.7 (8)
F1—C1—C2—C3	121.1 (5)	C3—C2—O1—Ni1	15.5 (6)
O1—C2—C3—C4	2.6 (8)	C1—C2—O1—Ni1	−164.6 (3)
C1—C2—C3—C4	−177.3 (4)	O4—Ni1—O1—C2	63.7 (3)
C2—C3—C4—O2	−4.0 (7)	O2—Ni1—O1—C2	−22.9 (3)
C2—C3—C4—C5	178.7 (4)	O3—Ni1—O1—C2	154.8 (3)
O2—C4—C5—C6	155.5 (4)	O6—Ni1—O1—C2	−112.5 (3)
C3—C4—C5—C6	−27.0 (6)	C3—C4—O2—Ni1	−14.1 (6)
O2—C4—C5—C8	−83.9 (5)	C5—C4—O2—Ni1	163.3 (3)
C3—C4—C5—C8	93.6 (5)	O4—Ni1—O2—C4	−65.2 (3)
O2—C4—C5—C7	33.8 (5)	O1—Ni1—O2—C4	23.5 (3)
C3—C4—C5—C7	−148.7 (4)	O6—Ni1—O2—C4	113.4 (3)
F4—C9—C10—O3	−42.5 (7)	O5—Ni1—O2—C4	−157.7 (3)
F6—C9—C10—O3	75.5 (6)	C11—C10—O3—Ni1	−3.3 (7)
F5—C9—C10—O3	−165.9 (5)	C9—C10—O3—Ni1	179.0 (3)
F4—C9—C10—C11	139.4 (5)	O4—Ni1—O3—C10	9.4 (3)
F6—C9—C10—C11	−102.6 (6)	O1—Ni1—O3—C10	−79.3 (3)
F5—C9—C10—C11	16.1 (7)	O6—Ni1—O3—C10	−169.2 (3)
O3—C10—C11—C12	−4.0 (9)	O5—Ni1—O3—C10	102.0 (3)
C9—C10—C11—C12	173.7 (5)	C11—C12—O4—Ni1	11.3 (7)
C10—C11—C12—O4	−0.2 (8)	C13—C12—O4—Ni1	−169.9 (3)
C10—C11—C12—C13	−178.9 (5)	O2—Ni1—O4—C12	166.5 (4)
O4—C12—C13—C14	11.2 (6)	O1—Ni1—O4—C12	75.8 (4)
C11—C12—C13—C14	−170.0 (4)	O3—Ni1—O4—C12	−13.9 (4)
O4—C12—C13—C16	−108.5 (5)	O5—Ni1—O4—C12	−100.9 (4)
C11—C12—C13—C16	70.3 (5)	O4—Ni1—O5—C17	39.8 (5)
O4—C12—C13—C15	130.8 (4)	O2—Ni1—O5—C17	126.6 (5)
C11—C12—C13—C15	−50.3 (6)	O3—Ni1—O5—C17	−51.1 (5)
F7—C18—C19—O7	178.4 (4)	O6—Ni1—O5—C17	−143.8 (5)
F9—C18—C19—O7	−60.8 (6)	C20—C19—O7—Ni2	5.1 (7)
F8—C18—C19—O7	57.5 (5)	C18—C19—O7—Ni2	−176.1 (3)
F7—C18—C19—C20	−2.7 (6)	O8—Ni2—O7—C19	−10.1 (3)
F9—C18—C19—C20	118.1 (5)	O9—Ni2—O7—C19	78.9 (3)
F8—C18—C19—C20	−123.6 (5)	O11—Ni2—O7—C19	−100.5 (3)
O7—C19—C20—C21	3.1 (8)	O12—Ni2—O7—C19	171.3 (3)
C18—C19—C20—C21	−175.6 (4)	C20—C21—O8—Ni2	−8.8 (7)
C19—C20—C21—O8	−1.3 (8)	C22—C21—O8—Ni2	171.3 (3)
C19—C20—C21—C22	178.6 (5)	O10—Ni2—O8—C21	−167.3 (4)
O8—C21—C22—C23	124.7 (6)	O7—Ni2—O8—C21	12.5 (4)
C20—C21—C22—C23	−55.2 (7)	O9—Ni2—O8—C21	−78.3 (4)
O8—C21—C22—C25	3.6 (7)	O11—Ni2—O8—C21	101.5 (4)
C20—C21—C22—C25	−176.3 (5)	C28—C27—O9—Ni2	−26.1 (7)
O8—C21—C22—C24	−115.0 (5)	C26—C27—O9—Ni2	153.3 (3)
C20—C21—C22—C24	65.1 (6)	O8—Ni2—O9—C27	−57.6 (3)
F10—C26—C27—O9	−52.4 (5)	O10—Ni2—O9—C27	32.3 (3)
F12—C26—C27—O9	66.9 (5)	O7—Ni2—O9—C27	−148.6 (3)
F11—C26—C27—O9	−173.4 (4)	O12—Ni2—O9—C27	120.5 (3)
F10—C26—C27—C28	127.0 (5)	C28—C29—O10—Ni2	8.3 (7)
F12—C26—C27—C28	−113.6 (5)	C30—C29—O10—Ni2	−173.8 (3)
F11—C26—C27—C28	6.1 (7)	O8—Ni2—O10—C29	63.7 (4)
O9—C27—C28—C29	−1.3 (8)	O9—Ni2—O10—C29	−25.3 (4)
C26—C27—C28—C29	179.3 (4)	O11—Ni2—O10—C29	154.1 (4)
C27—C28—C29—O10	12.1 (8)	O12—Ni2—O10—C29	−117.8 (4)
C27—C28—C29—C30	−165.8 (5)	O8—Ni2—O11—C34	44.0 (5)
O10—C29—C30—C32	−111.8 (6)	O10—Ni2—O11—C34	−45.9 (5)
C28—C29—C30—C32	66.2 (7)	O7—Ni2—O11—C34	135.0 (5)
O10—C29—C30—C33B	63.0 (16)	O12—Ni2—O11—C34	−134.1 (5)
C28—C29—C30—C33B	−119.0 (16)	O10—Ni2—O12—C35	31.0 (5)
O10—C29—C30—C31	11.8 (8)	O7—Ni2—O12—C35	−148.8 (5)
C28—C29—C30—C31	−170.2 (6)	O9—Ni2—O12—C35	−57.9 (5)
O10—C29—C30—C32B	−160.8 (11)	O11—Ni2—O12—C35	122.2 (5)

### Hydrogen-bond geometry (Å, °)

**Table:**

*D*—H···*A*	*D*—H	H···*A*	*D*···*A*	*D*—H···*A*
O5—H5A···O9^i^	0.82 (4)	1.87 (2)	2.692 (4)	175 (5)
O6—H6D···O7	0.84 (4)	1.89 (2)	2.701 (4)	164 (5)
O6—H6E···O12^i^	0.81 (4)	2.16 (2)	2.951 (5)	164 (5)
O11—H11A···O1	0.85 (5)	1.93 (5)	2.764 (4)	166 (5)
O12—H12A···O3	0.84 (2)	1.94 (2)	2.778 (4)	173 (5)
O12—H12B···O10	0.84 (2)	2.5 (1)	2.861 (4)	108 (11)

Symmetry codes: (i) −*x*+1, −*y*+1, −*z*+1.
